# Supplementary material for: Characterizing BOLD activation patterns in the human hippocampus with laminar fMRI
Source: Imaging Neurosci (Camb). 2025 Apr 8;3:imag_a_00532. doi: 10.1162/imag_a_00532 (PMC12319889; doi:10.1162/imag_a_00532)
Supplement: Supplementary Material [file imag_a_00532-supp.pdf]

# Supporting Information

## For “Characterizing BOLD activation patterns in the human hippocampus with laminar fMRI”

**Viktor Pfaffenrot, Antoine Bouyeure, Carlos Alexandre Gomes, Sriranga Kashyap, Nikolai Axmacher, and David G. Norris**

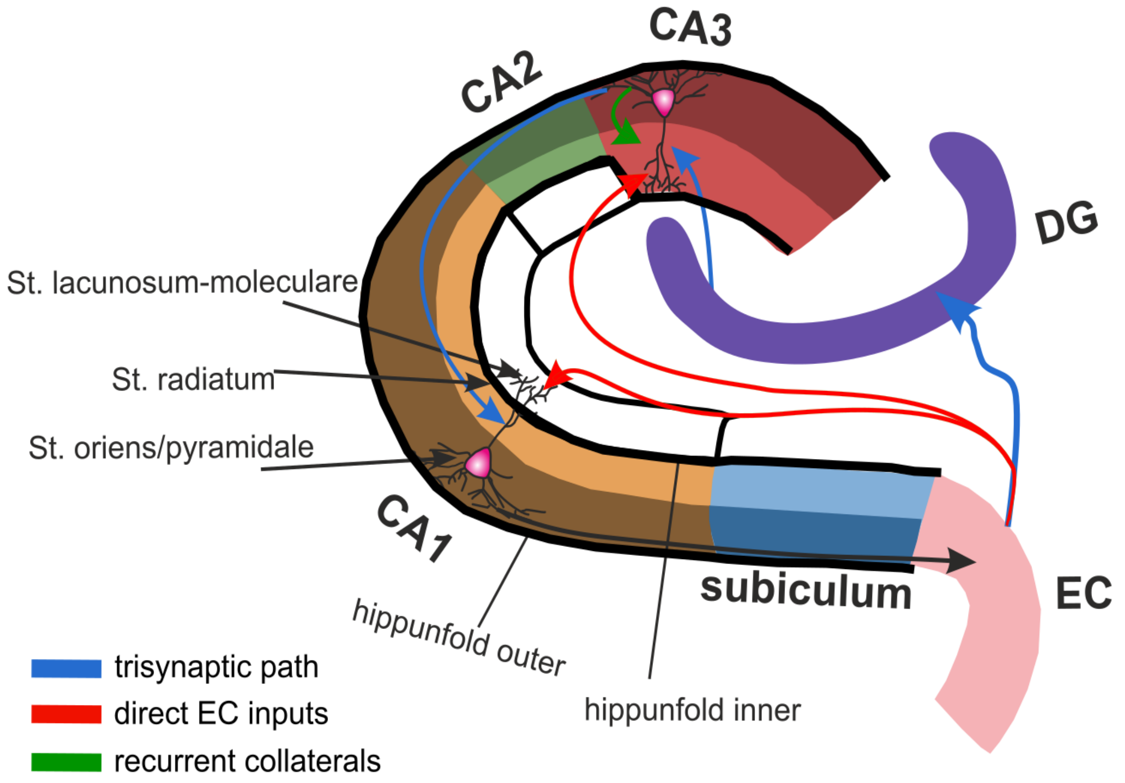


**Fig. S1: Neuronal pathways of the hippocampus.** Schematic depiction of the neuronal pathways of the hippocampus. Inner layers are in lighter colors, outer layers in darker colors. The St. radiatum and St. lacunosum-moleculare together form the SRLM (shown in white). The surfaces as given by hippunfold (DeKraker et al., 2022) are shown for reference. The orientation is the same as in Fig. 1D of the main manuscript.

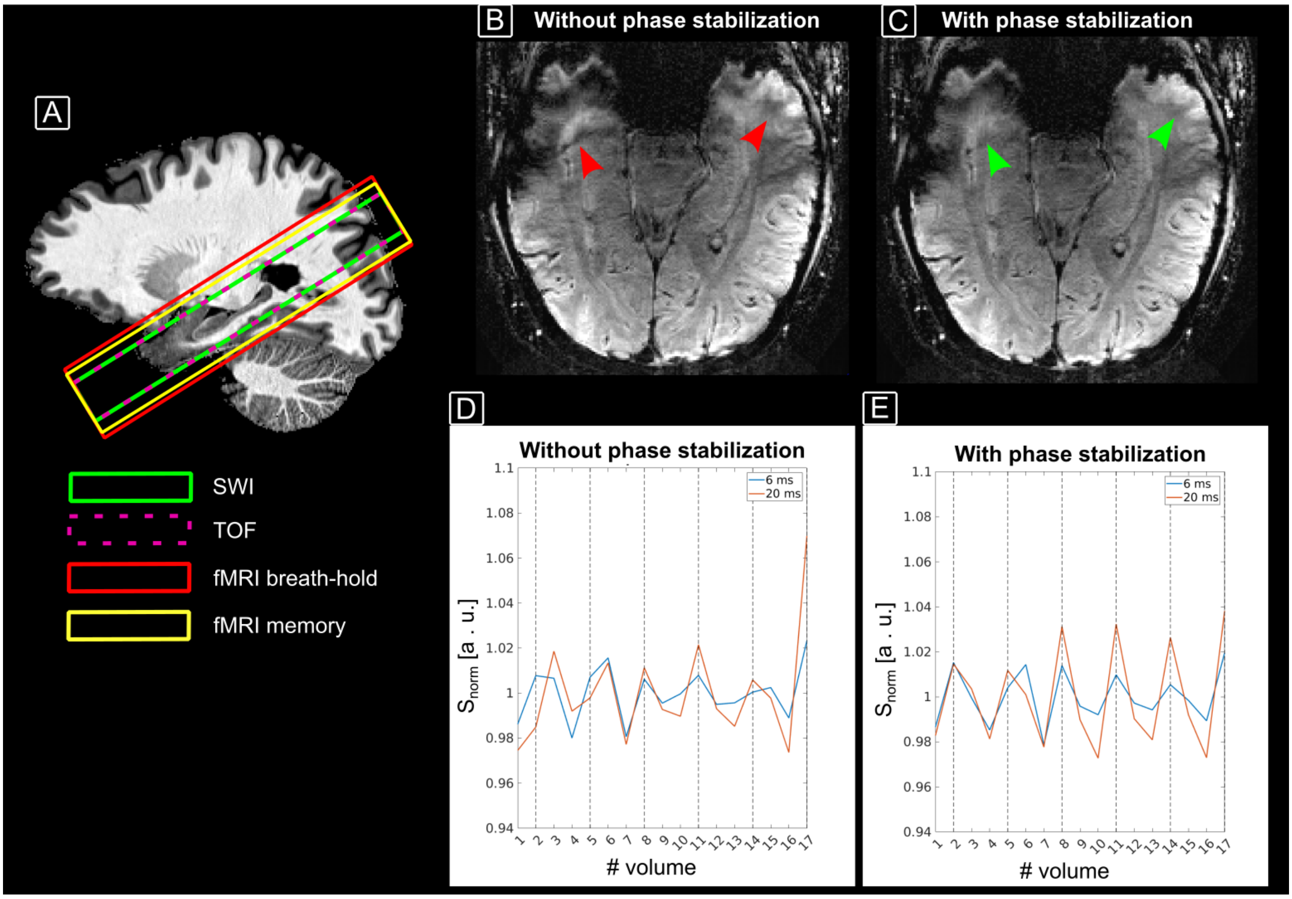


**Fig. S2: FOV placement and breath-hold signal stability. A** FOV placement of each MRI contrast with respect to the anatomical reference. **B-C** Images acquired during the inhalation period (transition phase) of the last echo (TE = 21 ms) without (**B**) and with (**C**) phase stabilization. $B_0$-induced artifacts (red arrows in **B**) are reduced with phase stabilization (green arrows in **C**). **D-E** Time series of the echo-normalized signal obtained from the inner surface of the hippocampus for TE = 6 ms (blue lines) and 20 ms (red lines). The dashed vertical lines correspond to an ‘active’ volume. With phase stabilization (**E**), the signal change is more consistent, and coincides well with the paradigm, especially at longer echoes.

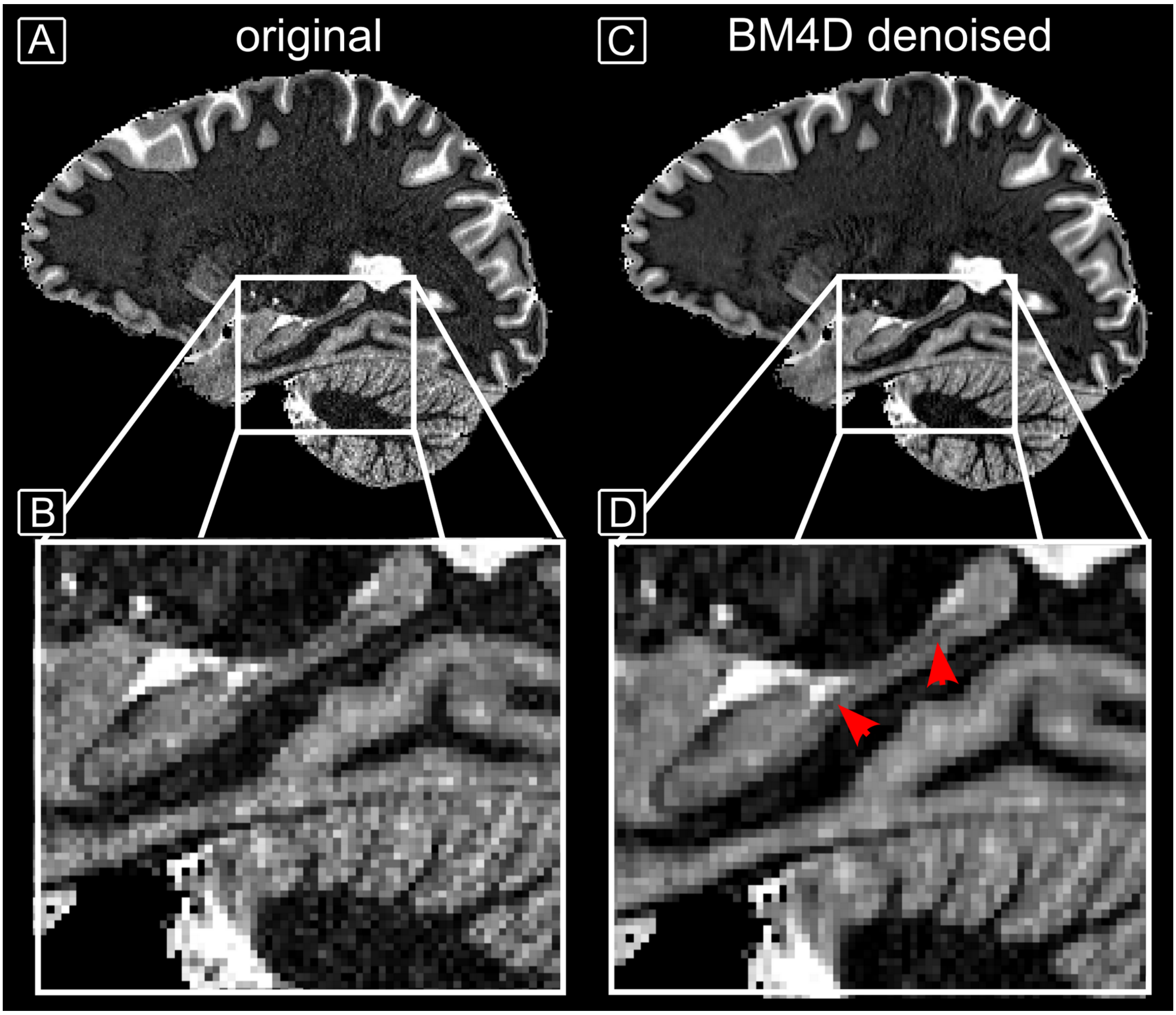


**Fig. S3: Denoising performance. A** Original $T_1$-weighted image (first inversion time of MP2RAGE, i.e. INV1). **B** Zoomed section in A showing the hippocampus and parts of the cerebellum as a region with typically low SNR. **C** INV1 after denoising using BM4D. Salt and pepper noise is strongly suppressed while fine-scale details are preserved (e.g. red arrow in D showing the vestigial hippocampal sulcus). **D** Zoome section in C.

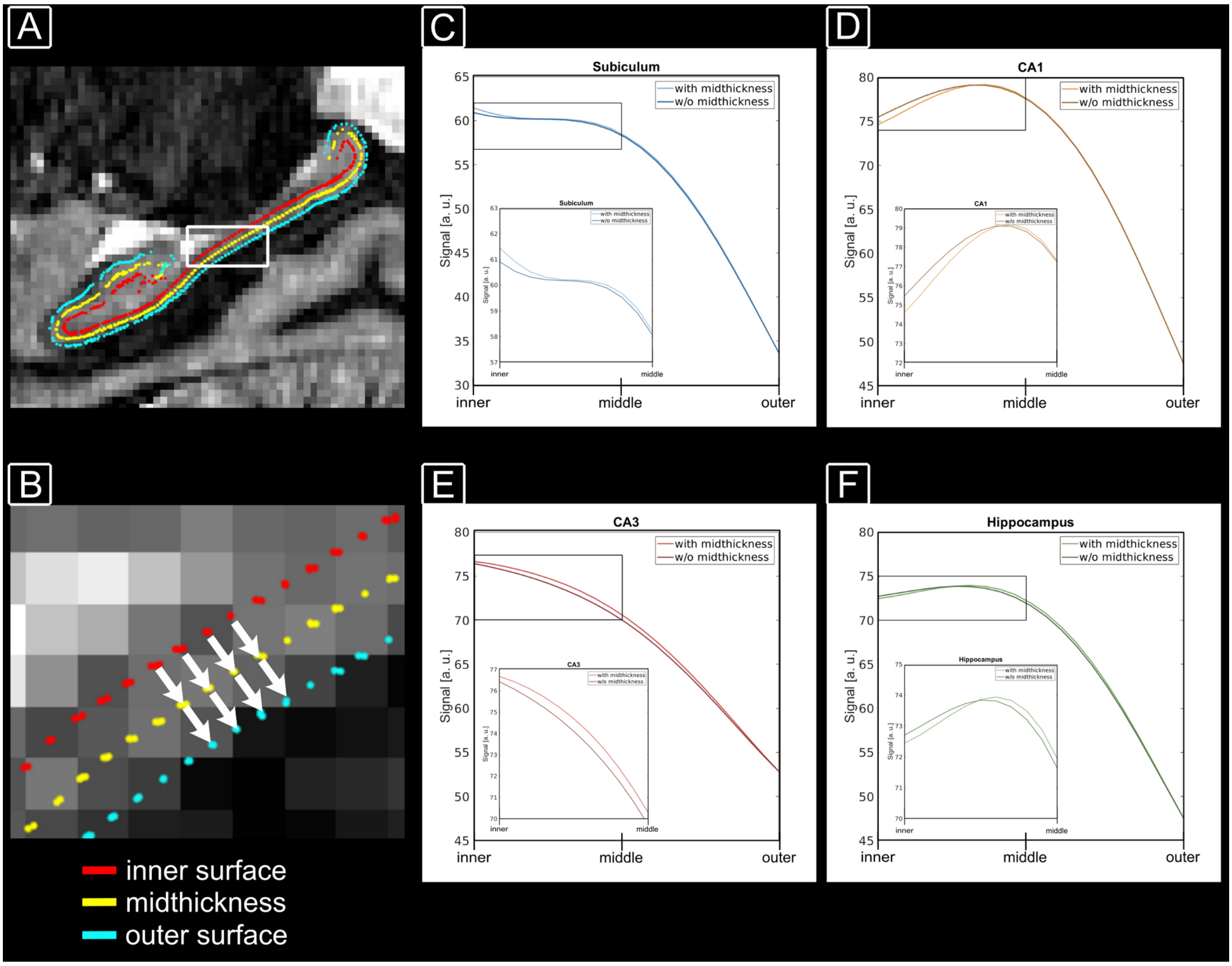


**Fig. S4: Hippocampus laminar sampling strategy. A** Surfaces obtained with hippunfold. **B** Zoomed section in **A**. Each vertex on the inner surface has a counterpart on the outer surface. For these vertex pairs, the signal is sampled, taking the middle surface as an anchor point into account (white arrows in **B**). This strategy is similar to that presented by Koopmans et al., 2011. **C-F** Signal sampled from the INV1 image of the MP2RAGE (**A-B**) for example subregions and for the entire hippocampus with and without taking the midthickness surface into account. Zoomed sections are shown as inlays. The sampling strategy essentially obeys the equivolume principle (Bok, 1929), but similar to the findings of Waehnert et al., 2014 the profiles do not strongly differ from those obtained with a pure equidistant sampling given the still relatively coarse resolution of typical laminar fMRI experiments.

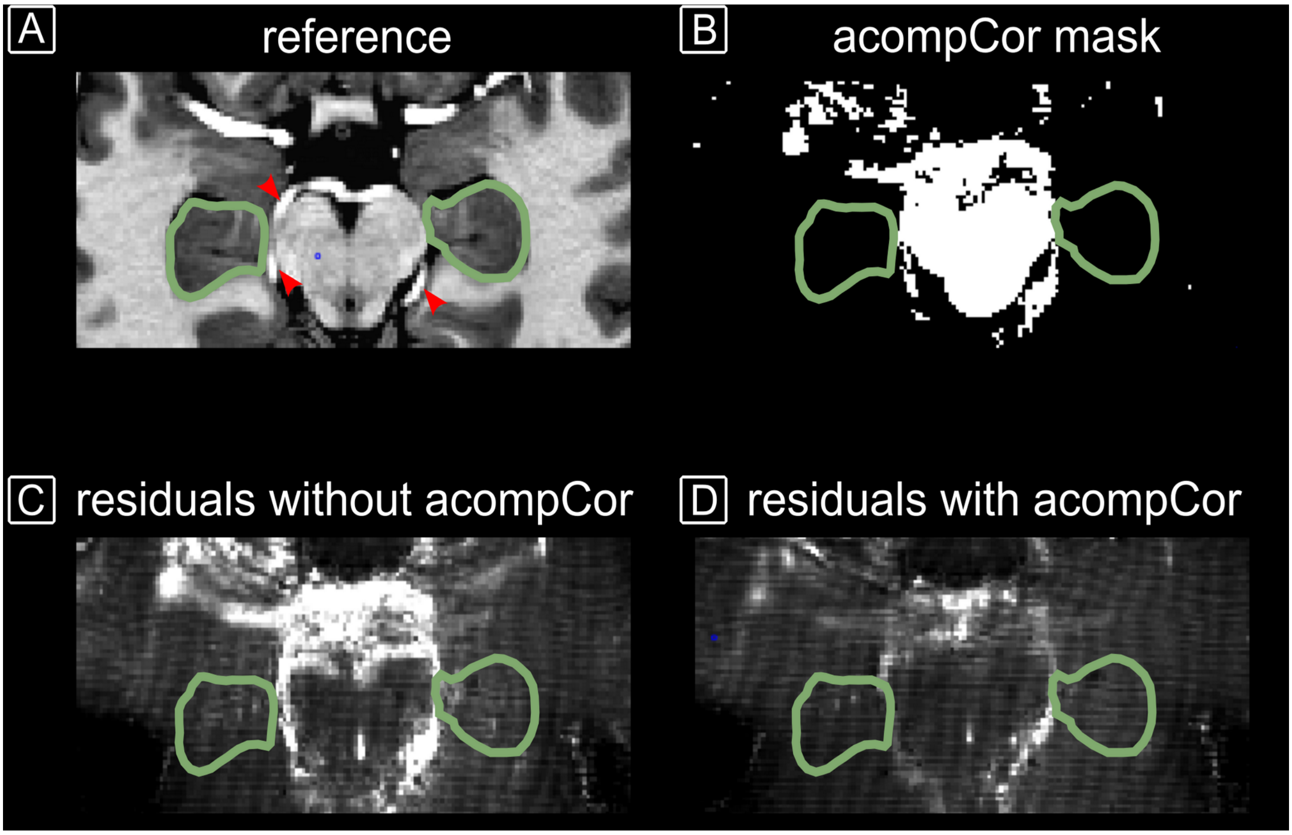


**Fig. S5: Physiological noise correction strategy.** Anatomical reference of an exemplary subject. The hippocampi are highlighted as green ROIs. Note the large arteries (red arrows) close to the hippocampus. **B** Mask used for acompCor regression. It contains the manually drawn brainstem white matter mask and a mask of high residuals as given by the first GLM. **C** Residuals of the first GLM. **D** Residuals after the second GLM using approx. 13 acompCor regressors. The residuals within the hippocampus reduced from 195 ± 76 to 170 ± 36.

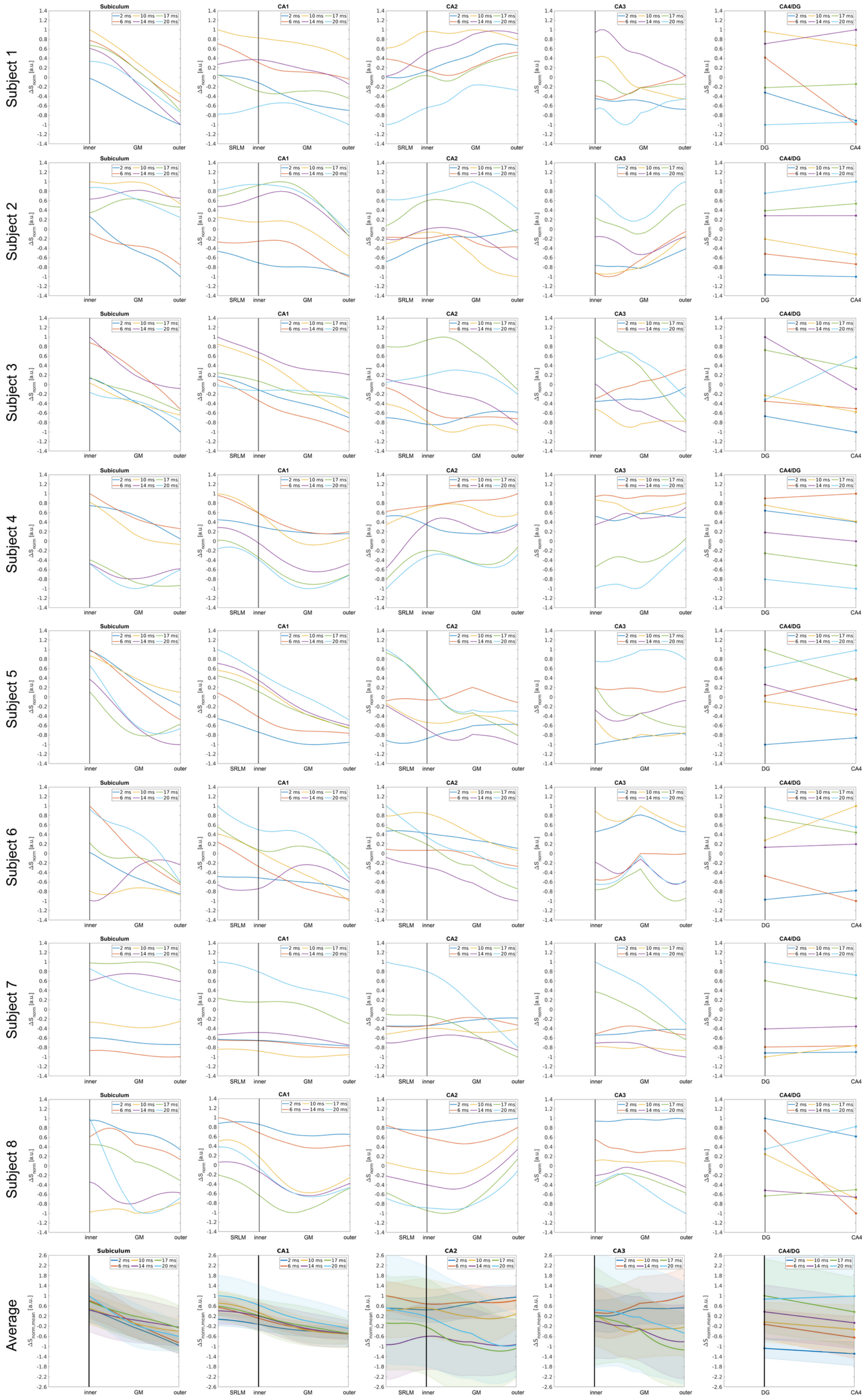


**Fig. S6: Single subject and subject-averaged multi-echo breath-hold results.** Profiles are shown for each subject and subfield. The shaded area in the average plots (bottom row) is the SEM.

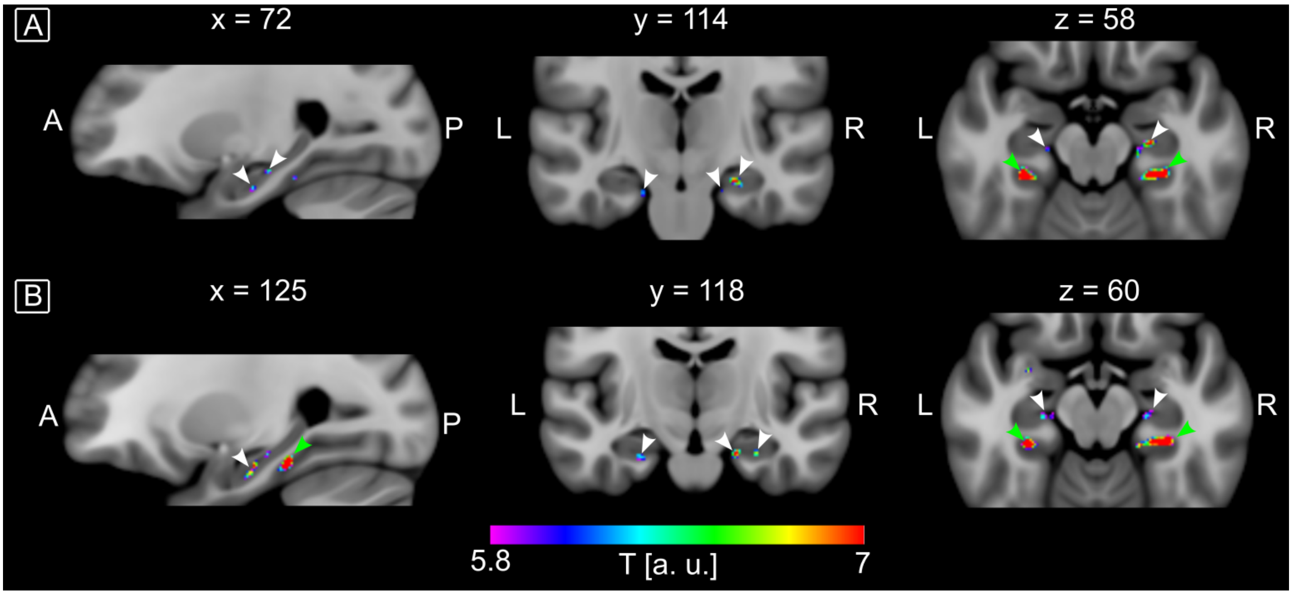


**Fig. S7: Voxel level group results for the memory > math contrast.** Maps of pseudo t-values at the group level as computed with the SnPM toolbox (p < .05, FWE-corrected, coordinates given in MNI space). The maps show the expected activation patterns for the hippocampus (white arrows, see also Fig. 1 in Leelaarporn et al., 2024) and the parahippocampal gyri (green arrows) when performing the autobiographical memory paradigm. The opposite contrast (math > memory) showed no significant voxels.

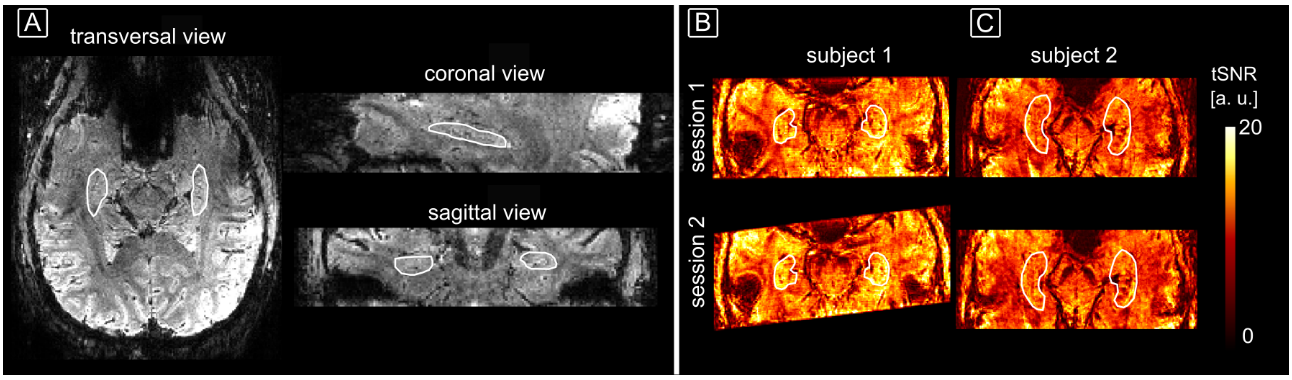


**Fig. S8: Autobiographical memory experiment: image quality. A** Example single volume of the first subject (in native space). The hippocampus is highlighted as white ROI. **B-C** tSNR maps of both sessions for subject 1 (**B**) and subject 2 (**C**) (in anatomical space). The tSNR in the hippocampus averaged over both hemispheres (white ROIs) was similar between sessions (Subject 1: 11.3 ± 3.2 for session 1, 11.4 ± 3.2 for session 2; Subject 2: 8.4 ± 2.5 and 8.6 ± 2.6, respectively).

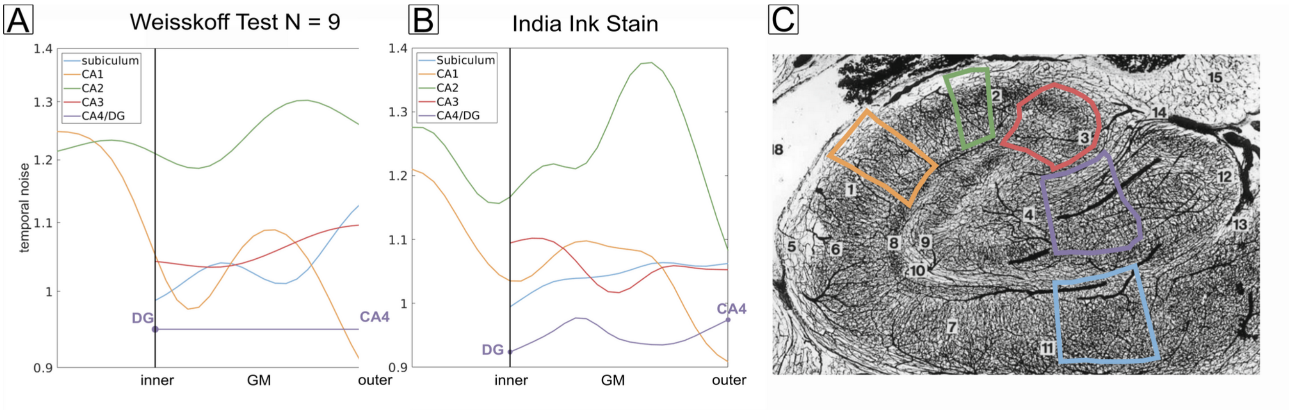


**Fig. S9: Comparison between profiles of physiological noise and microvascular density. A** Noise profiles in the physiological noise dominated regime (same as Fig. 6F of the main manuscript). We sampled the pixel intensity in the colored regions highlighted in **C** by adapting the approach as described in (**https://layerfmri.com/2018/07/19/2dlayers/#more-950**). The regions mark parts of the different subfields (image adapted from Duvernoy et al., 2013). **B** Obtained profiles smoothed to match the effective resolution of the main experiment. The profiles show a strong resemblance to those obtained from the Weisskoff test (A) indicating that the variation of physiological noise profiles could be caused by the underlying blood volume distribution.

**Table S1: Hippocampal GM thickness estimates**

| **Thickness [mm] (mean ± SD)** | **Subiculum** | **CA1** | **CA2** | **CA3** | **CA4** |
|---|---|---|---|---|---|
| Hippunfold (wo SRLM) | 1.37 ± 0.08 | 1.47 ± 0.08 | 1.16 ± 0.06 | 1.38 ± 0.08 | 1.91 ± 0.13 |
| Hippunfold (with SRLM) | 1.37 ± 0.08 | 2.21 ± 0.13 | 1.75 ± 0.08 | 1.38 ± 0.08 | 1.91 ± 0.13 |

## Analysis along the long axis of the hippocampus

The longitudinal axis has been shown to be one of the main mode of organization of the hippocampus (Poppenk et al., 2013). To investigate variations along the longitudinal axis of the hippocampus, we conducted additional analyses by dividing each subfield into head, body, and tail segments, i.e. corresponding to the anterior, middle and posterior parts of each subfield along the longitudinal axis of the hippocampus. This was done by manually segmenting each hippocampus into four longitudinal sections, following the methodology

described in Leelaarporn et al., 2024. We then combined the two anteriormost sections (head and anterior body) to obtain the classical three-thirds head, body, and tail longitudinal division of the hippocampus. Then, we computed the intersection between these longitudinal masks and the subfields masks, obtaining, for each subject, the head, body and tail longitudinal sections of each subfield. We used these masks to conduct layer fMRI analyses that assess variations of BOLD signal along the cortical depth of each subfield, separately for the head, body and tail longitudinal section of these subfields, based on the breath-hold data, and on the autobiographical memory data using both the memory > math and pre > post contrasts.

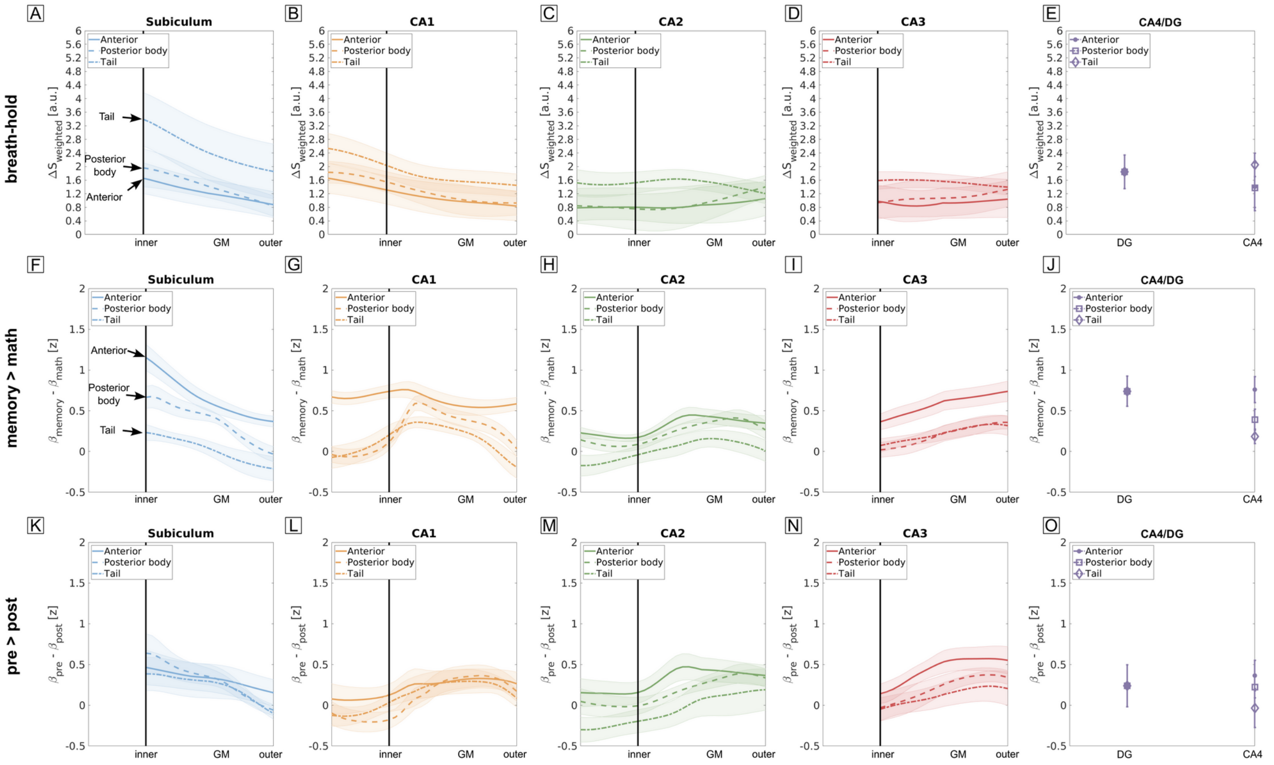


**Fig. S10: Layer-specific activity patterns across the longitudinal axis of hippocampal subfields. A-E** Breath-hold responses showing vascular bias patterns in subiculum, CA1-4, and DG. Note the prominent inner-surface bias in subiculum tail and consistent bias in CA1. **F-J** Memory>math contrast revealing subfield-specific longitudinal variations, including anterior-dominated responses in subiculum and distinct middle-layer peaks in posterior CA1. **K-O** Pre>post contrast showing consistent longitudinal patterns with notable outer-layer activation in CA1 opposing vascular bias direction. Data presented as mean ± SEM across participants, with anterior (solid), posterior body (dashed), and tail (dot-dashed) segments shown separately. Inner/outer designate surface direction.

The breath-hold experiment (Supplementary Figure S10A-E) revealed distinct patterns of bias across subfields and longitudinal segments: In the subiculum (A), a prominent bias toward inner surfaces was observed, with the tail showing the strongest effect. CA1 (B) displayed a similar inner-surface bias that was consistent across all longitudinal segments. CA2 (C) and CA3 (D) showed relatively uniform responses across layers, while CA4/DG (E) was assessed at specific anatomical points.

In the memory > math contrast (Supplementary Figure S10F-J), we observed subfield-specific longitudinal variations. The subiculum (F) showed the strongest activation in its anterior portion (head), with activity decreasing toward outer layers across all longitudinal segments. CA1 (G) revealed a distinct pattern, where body and tail segments peaked in middle layers (GM), contrasting with the head segment profile. Notably, the middle-layer peak cannot be explained by the vascular bias observed in the breath-hold data (B). CA2 (H) showed moderate activation across layers, while CA3 (I) exhibited a gradual increase toward outer layers, most pronounced in the head. CA4/DG (J) showed variable responses across longitudinal segments.

The pre > post contrast (Fig. S10K-O) revealed different patterns. Activity in the subiculum (K) decreased toward outer layers across all longitudinal segments. CA1 (L) showed increased signal change toward outer layers, notably in the opposite direction of the breath-hold bias, suggesting a neural rather than vascular origin. CA2 (M) and CA3 (N) displayed modest layer-specific effects, while CA4/DG (O) showed consistent activation across segments.

## Bibliography


Bok, S. T. (1929). Der Einfluß der in den Furchen und Windungen auftretenden Krümmungen der Großhirnrinde auf die Rindenarchitektur. *Zeitschrift Für Die Gesamte Neurologie Und Psychiatrie*, *121*(1), 682–750. https://doi.org/10.1007/BF02864437/METRICS

DeKraker, J., Haast, R. A. M., Yousif, M. D., Karat, B., Lau, J. C., Köhler, S., & Khan, A. R. (2022). Automated hippocampal unfolding for morphometry and subfield segmentation with HippUnfold. *eLife*, *11*, e77945. https://doi.org/10.7554/eLife.77945

Duvernoy, H. M., Cattin, F., Risold, P. Y., Vannson, J. L., & Gaudron, M. (2013). *The human hippocampus: Functional anatomy, vascularization and serial sections with MRI* (4th ed.). Springer Berlin, Heidelberg.

Koopmans, P. J., Barth, M., Orzada, S., & Norris, D. G. (2011). Multi-echo fMRI of the cortical laminae in humans at 7 T. *NeuroImage*, *56*(3), 1276–1285. https://doi.org/10.1016/j.neuroimage.2011.02.042

Leelaarporn, P., Dalton, M. A., Stirnberg, R., Stöcker, T., Spottke, A., Schneider, A., & McCormick, C. (2024). Hippocampal subfields and their neocortical interactions during autobiographical memory. *Imaging Neuroscience*, *2*, 1–13. https://doi.org/10.1162/IMAG_A_00105

Poppenk, J., Evensmoen, H. R., Moscovitch, M., & Nadel, L. (2013). Long-axis specialization of the human hippocampus. *Trends in Cognitive Sciences*, *17*(5), 230–240. https://doi.org/10.1016/j.tics.2013.03.005

Waehnert, M. D., Dinse, J., Weiss, M., Streicher, M. N., Waehnert, P., Geyer, S., Turner, R., & Bazin, P. L. (2014). Anatomically motivated modeling of cortical laminae. *NeuroImage*, *93*, 210–220. https://doi.org/10.1016/J.NEUROIMAGE.2013.03.078
